# The Leg Wound of King Philip II of Macedonia

**DOI:** 10.7759/cureus.2501

**Published:** 2018-04-18

**Authors:** Nicholas Brandmeir, Russell Payne, Elias Rizk, R. Shane Tubbs, Juan Luis Arsuaga, Antonis Bartsiokas

**Affiliations:** 1 Department of Neurosurgery, West Virginia University School of Medicine/Ruby Memorial Hospital; 2 Department of Neurosurgery, Penn State Milton S. Hershey Medical Center, Hershey, USA; 3 Neurosurgery, Seattle Science Foundation; 4 Departamento De Paleontología, Facultad De Ciencias Geológicas, Universidad Complutense de Madrid; 5 Laboratory of Physical Anthropology, Department of History and Ethnology, Democritus University of Thrace

**Keywords:** philip ii, alexander the great, knee granuloma, joint granuloma, peroneal nerve injury

## Abstract

Objective

King Philip II, the father of Alexander the Great, suffered a penetrating wound to the leg from a spear that left him severely handicapped. His skeletal remains represent the first and only case of an injury from ancient Greece that can be directly compared to its historical record. The objective of the study was to confirm the identity of the male occupant of Royal Tomb I in Vergina, Greece as Philip II of Macedonia by providing new evidence based on anatomical dissection and correlation with the historical description of the wounds.

Methods

Radiographs and photographs of the leg in Royal Tomb I in Vergina were examined. Anatomical dissection of a cadaver with a reconstructed wound similar to Philip’s was also completed to identify associated soft-tissue injuries.

Results

The left leg was penetrated by an object at the knee which resulted in joint diastasis, external rotation of the tibia, knee ankylosis, and formation of a granuloma around the related object. This caused massive trauma to the joint but spared the popliteal artery. This resulted in ligamentous injury as well as injury to the peroneal nerve and probably the tibial nerve, resulting in a complete palsy of those nerves.

Conclusion

This evidence exactly matches the historical sources and shows conclusively that the leg and Tomb I belong to Philip II. The anatomic and archaeologic evidence also serve as independent verification of some of the historical record of that period, better enabling scholars to judge the reliability of various texts. Furthermore, it gives invaluable information about surgical practices in ancient Greece according to Hippocratic methods and their outcomes. Finally, this sheds new light on the occupants of Royal Tomb II including the fact that the armor recovered there may have belonged to Alexander the Great.

## Introduction

Historical and archaeological background

Philip II of Macedonia was the most powerful man of his age. His career was characterized by the unification of ancient Greece and by the preparation of his son, Alexander the Great’s conquest of the Persian empire.

Throughout his life, Philip II exposed himself to danger. Demosthenes, who had seen Philip II when he paraded victoriously through Athens, said that he lost an eye to an arrow, suffered a fractured clavicle, and suffered a severe leg wound after being ambushed [1-2].

The Great Tumulus of Vergina, Greece was discovered as the burial site of the Macedonian Kings by Andronikos in 1977. The identification of the adult male in Royal Tomb I in Vergina as Philip II [3] confirmed the earlier hypothesis of Borza [3-5]. No other case from the ancient world provides such a complete historical record of an injury along with the associated remains.

Previous studies of war-time trauma from ancient Greece have focused entirely on historical and pseudohistorical accounts, especially the campaigns of Alexander the Great [6-7] and The Iliad [8-9]. Such accounts are limited because they have been translated and passed down through multiple authors. Despite these limitations, this evidence shows the ancient Greeks were familiar with limb injuries and neurovascular complications [8].

Traumatic injuries of the knee and related nerves

Modern examinations of knee injuries indicate that a high-energy impact is necessary to cause traumatic dislocation. This often results in multi-ligamentous injury and can also cause neurovascular injury [10]. The most common neurovascular structures injured in these events are the common peroneal nerve and popliteal artery [11-12]. Injuries of the common peroneal nerve have an incidence of 5%-40% in patients with a knee dislocation [10,11,13-15]. The lateral portion of the common peroneal nerve near the fibular head is most vulnerable to trauma [10]. Fewer than 40% of patients in modern series enjoy a functional recovery [14]. The mechanism of nerve injury is often a stretch lesion to the posterolateral aspect of the knee, although direct trauma can be involved in penetrating injuries [11,16]. The deeper location of the tibial nerve offers some protection from injury [10]. Tibial nerve injury in the setting of knee dislocation is always associated with concomitant common peroneal injury [16]. Its incidence has been reported as 53.6% in those with peroneal nerve injury from knee dislocation. The mechanism of injury is thought to be stretch or compression at the soleal sling [16].

Vascular injury is a common complication of traumatic knee injuries. Popliteal artery injuries, even with optimal care result in amputation 36% of the time [17]. When the artery cannot be repaired, amputation rates approach 72.5% [18]. Even today, popliteal artery injury represents a threat to life and limb. Injury of the popliteal vein can result in severe venous congestion and compartment syndrome that can also threaten limb viability [10].

Objectives

This case is the only example from ancient Greece and the world of an ancient injury with a contemporaneous historical description and identified remains [1]. This represents a unique and valuable addition to the history of medicine. The goal of this study was to use new archaeological anatomic evidence along with careful anatomic dissection combined with modern medical knowledge as well as an analysis of the historical record and historical medical practices to confirm the identity of an important archaeological specimen.

## Materials and methods

The historical accounts of Philip II’s leg injury were reviewed along with accounts related to medical treatment [1,2,19-20]. The fused leg from Tomb I was inspected visually with a magnifying glass and documented with macro-photography. The leg was then examined with computed tomography (CT) scan and plain radiographs. Three-dimensional (3-D) reconstructions of the CT scan were made with 3D slicer (3D Slicer, www.slicer.org) and 3-D modeling was done with 3D Builder (Microsoft Inc. Redmond, WA, USA). Anatomic dissections were carried out on the left leg of a well preserved male cadaver. Dissections were documented with macro-photography. X-rays were obtained with a fluoroscope (General Electric, Boston, MA, USA).

## Results

The ancient sources for the leg/knee wound of Philip II are consistent. The wound was in the thigh/leg and was nearly fatal [1]. Philip II and his army had prosecuted a successful war against the Scythians who lived northwest of the Danube. On the way back to Macedonia, the Triballoi, a Thracian tribe, ambushed Philip’s column after he refused to share the spoils. The probable purpose of the attack was to allow the Triballoi to seize the booty. Philip was severely wounded and the Triballoi attack succeeded in its aims [2].

**Figure 1:**
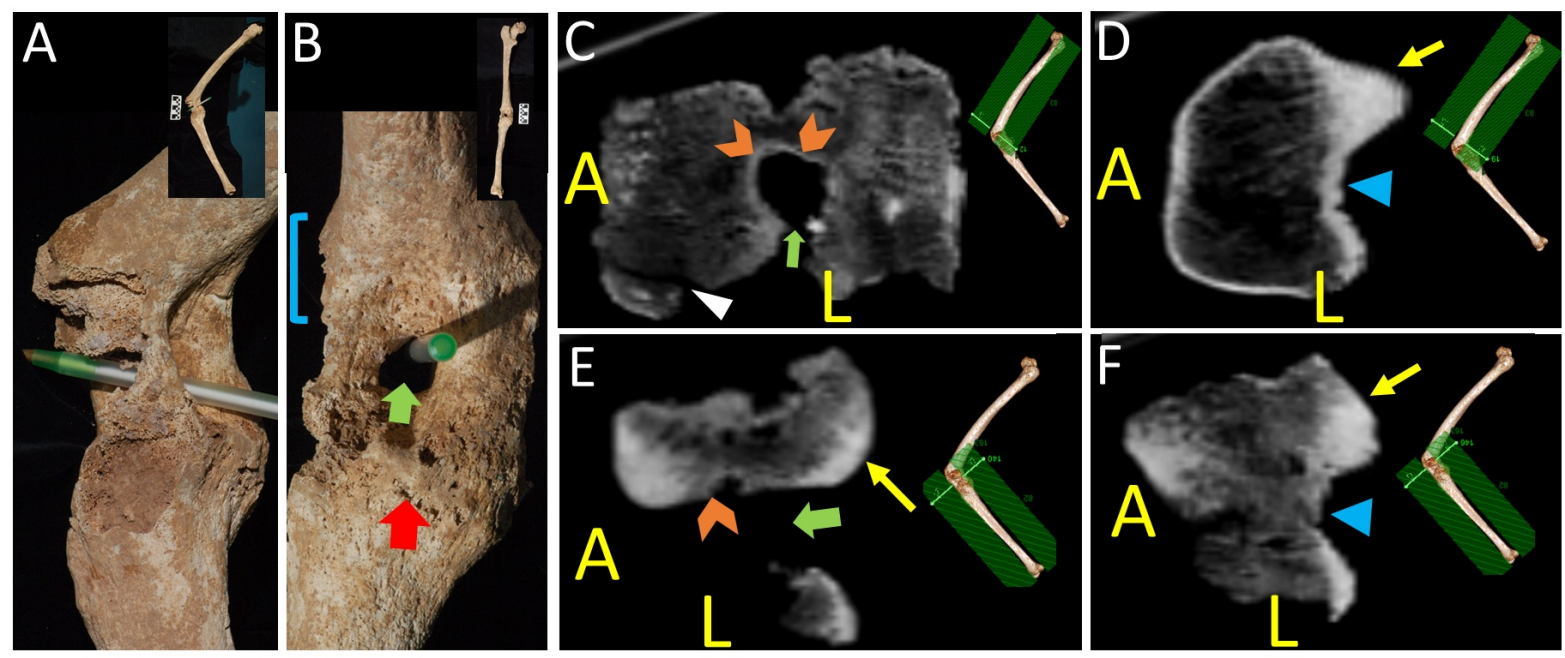
Views of Philip’s Leg A: Lateral view of the knee joint with a pen demonstrating the persistent space from the wound tract and foreign body granuloma and surrounding ankylosis. B: Posterior view of the knee joint along with the persistent space from the wound and foreign body granuloma and surrounding ankylosis. C: Axial CT scan through the injured joint space oriented perpendicular to the long axis of the femur. White triangle arrowheads point to areas of post-mortem damage identified by lack of compact bone on the surface and fracture lines separating continuous channels in the cancellous bone. Orange chevron arrowheads demonstrate reactive sclerotic bone on the walls of the granuloma. Note the osteolytic nature of the lesion with some sclerotic changes around the hole typical of lesions resulting from foreign body granulomas. D: Axial CT scan through the femoral condyles oriented perpendicular to the long axis of the femur. The yellow arrow represents normal cortical bone on the surface of the femoral condyles, whose shape can be appreciated. E: Axial CT scan of the injured joint space at a level inferior to the intercondyloid fossa oriented perpendicular to the long axis of the tibia. The hole can be seen along its major axis (green arrow). The difference between the abnormal bone lining the hole (orange chevron) and the normal cortical bone along the inferior limit of the femoral condyle (yellow arrow) can easily be distinguished. F: Axial CT scan of the joint space at the level of the intercondyloid fossa oriented perpendicular to the long axis of the tibia. The yellow arrow represents normal cortical bone on the femoral condyles, whose shape can be appreciated. Note that the bone surrounding the intercondyloid fossa is evenly corticated, differentiating it from the granuloma space. Labels - Brackets: femoral condyles, Red arrow: Tibial plateau, Green block arrow: wound tract, Blue triangle arrow: Intercondyloid fossa, A: Anterior, L: Left

The wound was inflicted by a cavalryman or foot soldier with a spear. The weapon was described by the Roman historian Justin as a sarissa [1]. The wound is described as penetrating Philip’s leg and killing his horse beneath him, causing his men to fear for his life [1-2]. Thus, the wound was delivered from a lateral/posterolateral direction since a primary anterior-posterior (or posterior-anterior) direction would have spared his horse and would probably have severed his popliteal artery causing probable limb loss and death. Anatomical examination of the remains demonstrates a severe traumatic injury to the left knee (Figure 1). The joint is flexed at 79° and ankylosed [3] across both epicondyles at a distance to the tibial plateau. There is a persistent hole in the joint space that represents the location of a foreign body granuloma from a penetrating object. Evidence that granulomas can form around wooden foreign bodies (including the surrounding ankylosis) is extensive [21-22]. Such granulomas were a known complication of retained foreign bodies from trauma [23]. As a group, these injuries indicate a severely crippling wound.

**Figure 2:**
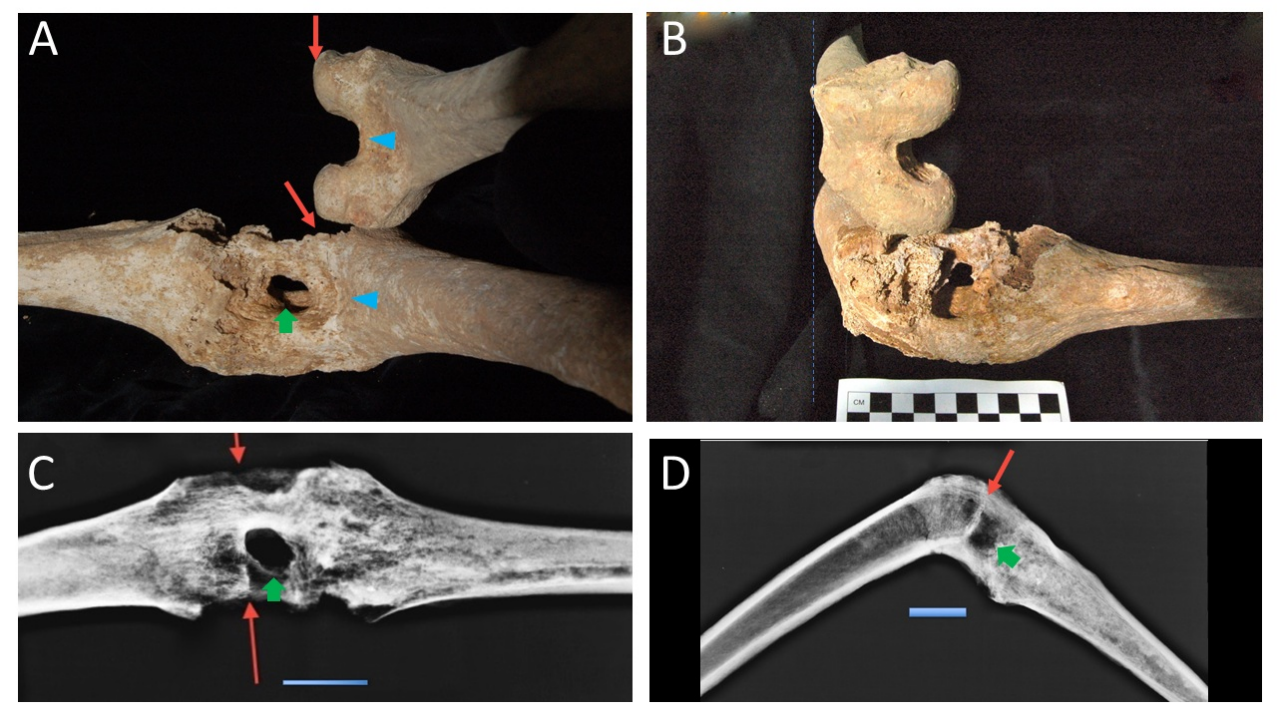
Demonstration of the Femoral Condyles and Joint Space Granuloma A: Posterior view of the specimen (below) with a sex and length match sample showing the condyles above it in parallel. B: En face view of the femoral condyles demonstrating the separation of the intercondyloid fossa from the wound tract. The dashed line to the left shows parallel structures. Blue line is the anterior femoral surface; note how the hole in the bone is distal to the intercondyloid fossa. C: posterior-anterior projection X-ray of the knee wound showing the inferior limit of the femoral condyles (red arrows) and wound tract granuloma space (green arrow). D: Lateral X-ray of the knee showing the inferior limit of the femoral condyles (red arrow) and the joint space granuloma (green arrow). Labels - Red skinny arrow: inferior limit of the femoral condyles, Green block arrow: joint space granuloma from wound tract, Blue triangular arrowhead: Intercondyloid fossa. Scale = 5 cm in C and D.

**Figure 3:**
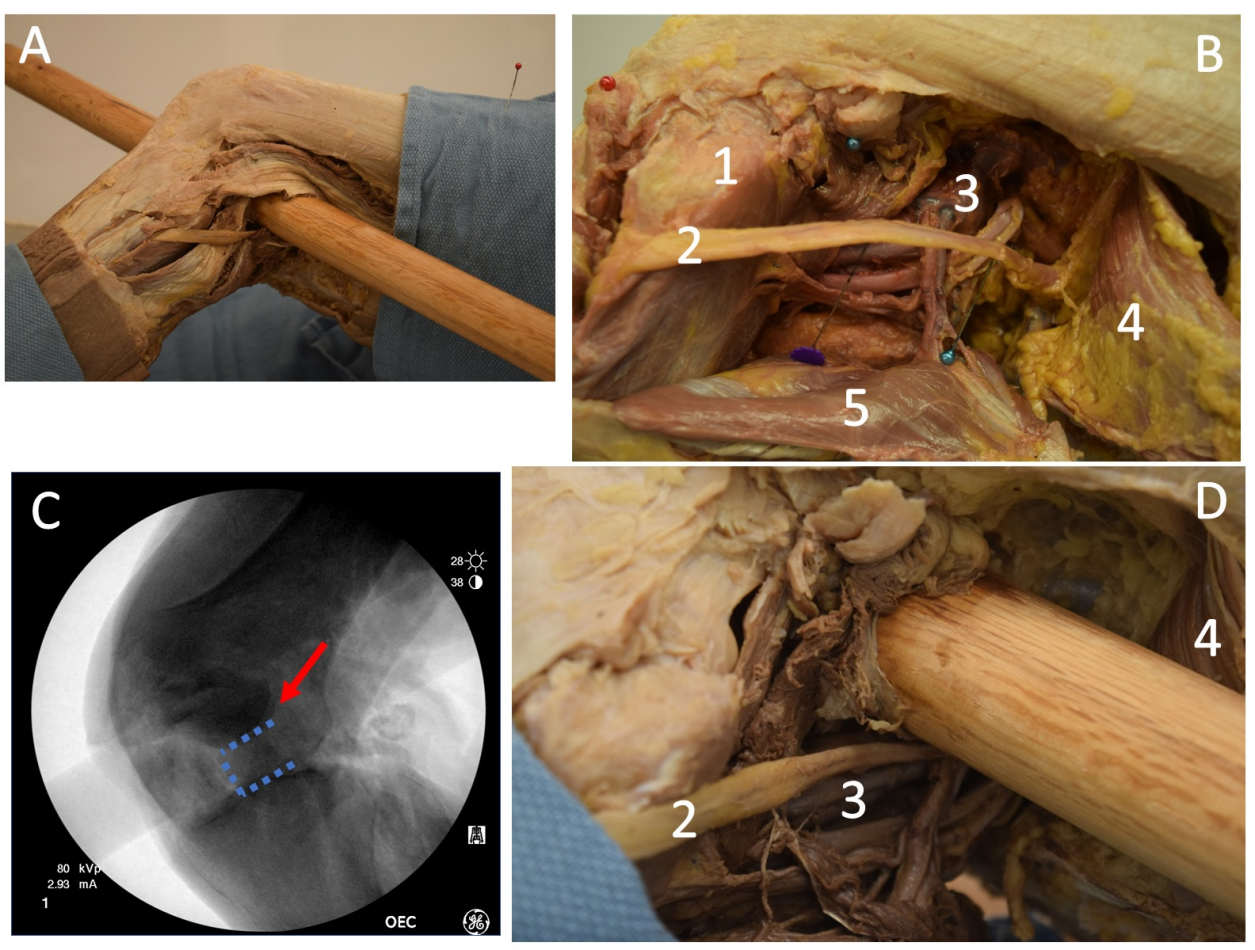
Cadaveric Dissection Recreating Philip’s Injury A: View of an example trajectory. Note how the spear enters medially to the long head of the biceps femoris and exits in the joint space between the femoral condyles. B: Close-up view of the dissection with anatomic structures labeled. C: Lateral oblique X-ray of cadaver specimen with a recreated injury. Note the joint diastasis similar to the knee of the specimen from Tomb I as well as the external rotation of the tibia. The penetrating foreign body can be seen as well. The red arrow represents the medial femoral condyle, the dashed bracket shows the joint space. D: Close-up view of the lateral aspect of the knee and popliteal fossa with the spear inserted. Note how the spear must either divide or radically displace the peroneal nerve to achieve this trajectory and also how this trajectory spares the large vessels of the popliteal fossa. Labels - 1: Fibular head, 2: Peroneal nerve, 3: Popliteal artery and vein, 4: Biceps femoris, 5: Gastrocnemius.

Radiographs of Philip’s leg were used to delineate the borders of the femoral condyles, the tibial plateau, and the joint space. The bony growth, especially on the lateral surface of the knee, was more exuberant during Philip’s life but appears limited because of post-mortem damage (Figure 1). The CT scans also support that the gap in the joint ankylosis is secondary to a foreign body granuloma. The radiographic characteristics of foreign body granulomas are like those of Philip’s leg. The salient features are the osteolytic character of the lesion and surrounding sclerosis (Figure 1). Histologic sections of foreign bodies that produced similar osteolytic lesions have demonstrated granulomatous change [21]. The radiographs (Figure 2C), as well as the comparison to the other left male femora of similar length (Figures 2A-2B), demonstrate that the inferior border of the condyle stops short of the main portion of the hollow lesion (Figure 2C), putting it in the joint space (Figure 2). Using a 3D reconstruction of the bones, and 3D modeling, we predicted a spear trajectory that would have produced the lesion seen in the joint space. An anatomical study of the left knee of a well preserved male cadaver was done to determine if our hypothesized injury was consistent with the wound that a lance would cause along that trajectory (Figure 3). The trajectory started just medial to the biceps femoris tendon and continued anteriorly through the joint space, exiting in the midline. Penetrating the joint space with the spear caused both a joint diastasis as well as external rotation of the tibia. In the dissection specimen, the soft tissue of the posterior compartment had been released. Without this, rotational forces on the tibia would presumably have been much stronger because of the restrained movement of the joint in the superior-inferior (proximal-distal) direction by the surrounding soft tissue. In our dissection, penetration of the joint was enough to cause a similar rotation of the tibia, but the collapse of his horse on top of him could have contributed to the distraction of the joint and rotation of the tibia. This trajectory matched the bony evidence as well as the historical descriptions of the wound under study. In the cadaver, there was direct trauma to the peroneal nerve, but the popliteal artery and vein were spared. Lateral X-rays were taken (Figure 3C) and compared to the X-rays of the bony specimen from Tomb I (Figure 2). The X-rays of the cadaver closely matched the X-rays of the leg from Tomb I, providing evidence that the cadaveric dissection accurately represented the wound pathology of Philip II.

The attack would have caused a diastasis of the knee joint with a combined tibial and peroneal nerve injury and a ligamentous disruption. While the peroneal nerve could have been severed directly by the blow, it is unlikely that the tibial nerve suffered a sharp injury given its proximity to the popliteal artery. Any tibial injury was likely from the stretch on the nerve [16].

## Discussion

Philip II was always surrounded by his companions [19]. Among these were physicians and there are multiple examples of them treating Philip II and Alexander [2,19,24]. After sustaining the wound, Philip would have been brought to his physician for immediate treatment. The lance was probably still transfixed through Philip’s leg given the difficulty of withdrawing a stuck lance during battle. Our dissections confirmed the extreme force required to penetrate the knee with a lance and remove it.

There are no accounts of Philip’s medical treatment, but his treatment can be speculated upon. The first decision would have been whether the artery was involved and whether the limb was salvageable. As discussed above, the popliteal artery was probably spared because he survived with his leg intact, but bleeding could have been significant. The physician probably chose to withdraw the lance from the wound but may have decided to leave it in place until the bleeding was manageable. The United States’ Army had extensive experience with low-velocity penetrating trauma in the pre-antibiotic era during wars with Native Americans. The techniques used there are probably similar to those employed by Philip’s physician. Evidence for this is the similarity between the Spoon of Diocles and the Strong Forceps, both instruments for removing arrows from these disparate time periods. These American surgeons recommended, in the absence of major vessel injury, that the foreign material be removed as soon as possible to prevent infection [23,25]. There is some historical evidence that penetrating foreign bodies could be left in place even if they violated the skin, as in the case of Gregor Baci [26]. The medical corps of the Macedonian army at this time were surgically proficient [6]. Urgent removal of the lance is also suggested by the urgent removal of the arrow that struck Philip’s eye. Diodorus Siculus reports that Philip’s physician Critobulus of Cos removed the arrow expeditiously but infection still set in [24,27]. Even with an immediate removal, it is likely that the foreign material (i.e., splinters) would be retained in the wound tract and would produce a granuloma consistent with the evidence seen in the specimen.

After removing the lance, the wound would have been dressed. Dressings of penetrating injuries during this period usually consisted of linen or hempen bandages soaked in wine, vinegar or rose oil [25]. Hippocrates gives advice on dressing penetrating/open wounds of the knee [20]. There is ample evidence that Philip’s wounds were treated in a Hippocratic fashion, although none are conclusive. As mentioned above, Philip’s physician was from Cos [24], the home city of Hippocrates and his Asclepeion. Further, Hippocratic medicine was known to have been practiced and taught throughout Philip’s domain [28].

In 'On the Articulations', Hippocrates advises against actively reducing compound fractures involving the tibia or femur, as doing so was believed to make death inevitable [20]. In 'On Fractures', Hippocrates describes a technique for placing bandages to prevent movement of the limb/joint with specific advice for overcoming the greater forces of the leg [20]. According to these techniques, Philip’s leg would have been tightly bandaged in situ. Evidence for this is the angle of the knee in the specimen, which is consistent with that needed to ride a horse without stirrups, which is what Philip II was doing during the battle.

Hippocrates advises that any limb left in a dislocated position will lead to muscle wasting, fixed deformities and nerve damage [20] and this is congruous with modern experience [29]. In unstable joints, Hippocrates recommended a technique to promote fusion across the joint. Described for the shoulder, the technique involved rapidly penetrating the skin and joint space with a hot poker to promote scarring. Application of this technique to Philip’s knee could have encouraged the ankylosis in the specimen.

During the chronic phase, the knee probably suffered from post-traumatic stiffness. This led to a fixed deformity with scar formation across the diastasis of the joint [29]. The stiffness was probably exacerbated by the complete nerve injuries that accompanied the wound. Once fibrosis and contractures cause a fixed deformity, the motion segment cannot always be restored, even in modern medical practice [29]. This stiffness, combined with the granuloma likely contributed to the progression of cartilaginous change and eventual ankylosis in the joint.

For the last three years of his life, Philip would have suffered multiple sequelae from this wound. Philip probably suffered from complete peroneal and tibial nerve palsies that would have left him anesthetic over the lateral aspect of the shin, the dorsum, and the sole of the foot. It is likely that he had a complete foot drop with a loss of plantar flexion and the intrinsic muscles of the foot. Walking with a peroneal nerve injury and an immobile, flexed knee would have been extremely difficult and precluded any agile movement. This is consistent with the episode where Philip II chased Alexander in anger at a wedding feast but fell when he attempted to run [30]. The injury would have been disabling and prevented athletic activity. Speculations that the male from Tomb I was a grave robber who clambered into Tomb I are not consistent with the evidence presented here. In addition, grave robbers in looted tombs are found as complete skeletons as opposed to the partial skeletons of the occupants in Tomb I.

## Conclusions

It is clear that the leg discussed above belongs to Philip II. The granuloma in the knee joint reported here is evidence of a penetrating injury to the knee that can only be historically attributed to Philip II. No other historical Greek king was reported to have been handicapped in this way. Alternative hypotheses of the leg injury in question are not consistent with the evidence presented here. This conclusively confirms the earlier work which was based on all the bones found in Tomb I in Vergina.

This is the first and only example of an ancient wound for which there are both historical descriptions and the remains of the injured person. Together with the texts of Hippocrates this information can be used to create an accurate description of the injury. Moreover, they confirm high levels of surgical skill present in the ancient world and the long-standing position of the surgeon as a part of battlefield medicine.
